# Plasma Exosomal Proteomic Pattern of Epstein-Barr Virus-Associated Hemophagocytic Lymphohistiocytosis

**DOI:** 10.3389/fmicb.2022.821311

**Published:** 2022-04-06

**Authors:** Yan Xie, Li Yang, Pengfei Cao, Shen Li, Wentao Zhang, Wei Dang, Shuyu Xin, Mingjuan Jiang, Yujie Xin, Jing Li, Sijing Long, Yiwei Wang, Senmiao Zhang, Yang Yang, Jianhong Lu

**Affiliations:** ^1^Department of Hematology, National Clinical Research Center for Geriatric Disorders, Xiangya Hospital, Central South University, Changsha, China; ^2^Department of Microbiology, School of Basic Medical Science, Central South University, Changsha, China; ^3^National Healthcare Commission (NHC) Key Laboratory of Carcinogenesis, The Key Laboratory of Carcinogenesis and Cancer Invasion of the Chinese Ministry of Education, Cancer Research Institute, Central South University, Changsha, China; ^4^China-Africa Research Center of Infectious Diseases, Central South University, Changsha, China

**Keywords:** hemophagocytic lymphohistiocytosis, Epstein-Barr virus, exosomes, quantitative proteomics, biomarker

## Abstract

Epstein-Barr virus (EBV)-associated hemophagocytic lymphohistiocytosis (EBV-HLH) is a life-threatening syndrome, which is caused by EBV infection that is usually refractory to treatment and shows relapse. The development of new biomarkers for the early diagnosis and clinical treatment of EBV-HLH is urgently needed. Exosomes have been shown to mediate various biological processes and are ideal non-invasive biomarkers. Here, we present the differential plasma exosomal proteome of a patient with EBV-HLH before vs. during treatment and with that of his healthy twin brother. A tandem mass tag-labeled LC-MS technique was employed for proteomic detection. Gene Ontology and Kyoto Encyclopedia of Genes and Genomes analyses indicated that differential proteomic profiles were related to virus infection, coagulopathy, nervous system dysfunction, imbalance of immune response, and abnormal liver function. The candidate biomarkers were first identified in the patient’s plasma exosomes at different treatment and follow-up time points. Then, 14 additional EBV-HLH exosome samples were used to verify six differentially expressed proteins. The upregulation of C-reactive protein, moesin, galectin three-binding protein, and heat shock cognate 71 kDa protein and the downregulation of plasminogen and fibronectin 1 could serve as potential biomarkers of EBV-HLH. This plasma exosomal proteomic analysis provides new insights into the diagnostic and therapeutic biomarkers of EBV-HLH.

## Introduction

Hemophagocytic lymphohistiocytosis (HLH) is a severe life-threatening disease in which the immune system is seriously disordered (Zhang et al., 2021). The dysfunction of cytotoxic T lymphocytes and natural killer (NK) cells can lead to mononuclear macrophage systemic hyperplasia (Al-Samkari and Berliner, 2018). The excessive inflammatory reactions and bloodthirsty phenomena may ultimately lead to the multiple organ dysfunction syndrome (MODS) (Berlot et al., 2018). According to the inherited defects and external infectious factors, the disorder can be divided into primary HLH or secondary HLH (Filipovich and Chandrakasan, 2015). The primary HLH has been confirmed to be an autosomal recessive disorder and has related gene variants. The secondary HLH is usually caused by infection, rheumatism, and malignancy, and people of all ages have a risk of suffering from secondary HLH (Filipovich and Chandrakasan, 2015). The majority of patients with clinical HLH have secondary HLH with the viral infection, especially Epstein-Barr virus (EBV) infection, as one of the most common factors (Tabata YHibi et al., 2000). Although EBV is a ubiquitous herpesvirus that infects more than 90% of the human population, EBV infection causes a variety of life-threatening complications, which include HLH and several malignancies (Ko, 2015; Marsh, 2017). Its serious pathogenesis remains largely unclarified. The diagnosis of HLH can be confirmed by chart review according to the HLH-04 criteria (Song et al., 2019). Although the unique characteristics of HLH have been described in the diagnostic criteria, the diagnosis and treatment are still challenging because patients have multiple manifestations related to the multiple triggers (Jordan et al., 2011). In addition, compared with that of non-EBV-related HLH, the prognosis of EBV-related HLH is much worse (Song et al., 2019). At present, there is an urgent need for the early diagnosis and treatment of EBV-related HLH, which is the key for improving the survival rate of patients.

The exosomes are small (30–100 nm in diameter) extracellular vesicles that contain proteins, nucleic acids, and lipids, which can be shed by almost all cells. The emerging findings have suggested that exosomes play an important role in mediating viral infection, inflammation (Lee et al., 2016). Therefore, the exosomes are the promising candidates for developing disease biomarkers. However, there are currently no exosome-based biomarkers for EBV-HLH.

Recently, quantitative proteomics has become a useful tool for identifying potential protein biomarkers of various diseases. In this study, the plasma exosomes prior to and during the therapy from an adolescent patient with EBV-HLH and his twin brother (as a control) were used for the detection and analysis by tandem mass tag (TMT)-based quantitative proteomics, which could provide new insights into developing potential biomarkers for this uncommon but devastating disease.

## Materials and Methods

### Plasma Sample Collection

First, we collected blood samples from a patient with EBV-HLH at six different disease stages: the acute phase before treatment (T1), treatment for 17 days (T2), treatment for 28 days (T3) to 1 week after discharge (T4), 2 weeks after discharge (T5), and 3 weeks after discharge (T6). Then, we followed up this patient after discharge and collected plasma samples 1 year after the patient recovered. Interestingly, the patient has a healthy twin brother who lived together with him. Considering the strictness of the control, blood samples from the brother were collected and used as a control. In addition, blood samples from other patients with EBV-HLH and healthy controls were collected for an extensive validation. Plasma was extracted from the supernatant after the whole blood was centrifuged at 3,000 rpm for 5 min.

### Depletion of High-Abundance Albumin and Immunoglobulin G and Isolation of Exosomes From Plasma

The collected plasma samples were first treated with a ProteoPrep Blue Albumin and IgG Depletion kit (Sigma-Aldrich, St. Louis, MO, United States) according to the manufacturer’s instructions and then used to isolate exosomes according to our previous report (Liu et al., 2019).

### Transmission Electron Microscopy Assay

Transmission electron microscopy (TEM) assays were conducted as described previously (Liu et al., 2019). In brief, exosomes were purified and spotted onto grids (200 mesh) coated with formvar carbon and then fixed with 2% (wt/vol) paraformaldehyde for 5 min. The exosomes were stained with uranyl acetate, observed under a TEM (FEI Tecnai12) and imaged using a CCD camera.

### Protein Quantification and Coomassie Brilliant Blue Staining

The exosome protein concentration was measured with a BCA protein assay kit (Auragene Biotech, Changsha, China). After the exosome proteins were separated by SDS-PAGE, the protein bands were visualized by Coomassie brilliant blue staining.

### Proteomics of Exosomes

Liquid chromatography tandem MS (LC-MS/MS) analysis was performed for this exosome proteomic analysis, as previously reported (Que et al., 2020). In brief, the exosomal protein solution was reduced with dithiothreitol and then alkylated with iodoacetamide. After diluting the urea concentration of the protein sample to less than 2 M, trypsin was added for digestion two times. The trypsinized peptides were dissolved in 0.5 M TEAB buffer and labeled according to the TMT kit instructions. The labeled peptides were subjected to an NSI ion source for the ionization followed by tandem mass spectrometry (MS/MS) in Q ExactiveTM Plus (Thermo) coupled online to the ultraperformance liquid chromatography (UPLC). The MS/MS data were retrieved against the SwissProt Human database (20,317 sequences) using MaxQuant software (v1.5.2.8).

### Bioinformatic Analysis

Gene Ontology (GO) annotations at the proteomic level were derived from the UniProt-GOA database.^1^ The enriched GO analysis of annotated proteins was classified according to the cellular composition (CC), molecular function (MF), or biological process (BP). The Kyoto Encyclopedia of Genes and Genomes (KEGG) pathway database was used to annotate the enrichment pathways. The Venn diagrams web tool^2^ was used to compare the proteins identified with the ExoCarta database. The Search Tool for the Retrieval of Interacting Genes/Proteins (STRING) database and Cytoscape were applied to build a protein–protein interaction (PIP) network.

### Western Blotting

The exosomal proteins were first separated by SDS-PAGE and then transferred onto polyvinylidene difluoride membranes (Millipore, Billerica, MA, United States). After blocking with 5% (weight/volume) non-fat milk at 37°C for 1 h, the membranes were incubated with the relevant primary antibody at 4°C overnight, followed by incubation with the secondary antibody for 1 h at 37°C. All antibodies used in this study are listed in Supplementary Table 1.

### Statistical Analysis

After the gray value of the western blotting (WB) band was calculated by ImageJ, the normal group average gray value of each WB diagram was normalized to 1 to obtain the relative gray value of the EBV-HLH group. All statistical analyses were performed using GraphPad Prism 5. Differences between samples were determined using a two-tailed Student’s *t*-test, and *p* < 0.05 was considered significant.

## Results

### Case Presentation

A 15-year-old boy was admitted to the hospital with continuous fever for 5 days. On examination (T1), the patient’s temperature was 39.1^°^C, and he was accompanied by lymphadenectasis, splenomegaly, and anemia. Hemocytopenia, liver function, coagulation function, and inflammatory indices were abnormal. There were significantly high ferritin and multiple-organ failure appeared. Approximately 24% of pleomorphic lymphocytes and active hemophagocytosis were observed in the peripheral blood. EBV capsid antigen immunoglobulin M (IgM) was positive, cytomegalovirus IgM and herpes simplex virus type 1-IgM and immunoglobulin G (IgG) were negative. EBV DNA copy in plasma was 7.61 × 10^4^ IU/ml. According to the HLH-2004 diagnostic criteria (Henter et al., 2007), the patient was diagnosed with EBV-HLH. The patient’s laboratory test results are summarized in Table 1.

**TABLE 1:** Summary of the patients’ laboratory test results.

Inspection items	Reference range	Detection result
Hemoglobin (g/L)	120∼160	65
Reticulocyte (%)	0.5∼1.5	0.41
Leukocyte count (every 10^9/L)	4–10	3.21
Platelet count (every 10^9/L)	100–300	38
Aspartate aminotransferase (U/L)	0–40	473.3
Alanine aminotransferase (U/L)	0–40	348.2
Glutamine transpeptidase (U/L)	3–50	204.4
Alkaline phosphatase (U/L)	45–115	13.9
Total bilirubin (μmol/L)	1.71–17.1	26.0
Direct bilirubin (μmol/L)	1.71–7	17.0
Creatine kinase isozyme (U/L)	0–18	40.6
D-Dimer (mg/L)	0–0.55	12.725
Plasma fibrinogen (g/L)	2–4	1.5
Plasma prothrombin time (s)	10–14	16.9
Triglyceride (mmol/L)	0.56–1.7	3.89
Ferritin (μg/L)	15–200	>40,000

In the hospitalization period (T2–T3), ceftriaxone and ganciclovir were mainly used to prevent infection and antiviral treatment. ɣ-globulin and dexamethasone were given for immunomodulatory treatment, and etoposide was used for chemotherapy. During the treatment, the patient’s condition gradually improved, and the EBV-DNA of the patients was lower than the lower limit of detection in the T3 stage.

After 1.5 months of treatment, the patient was discharged (T4–T6). The patient had a normal body temperature, no fever and cough, stable liver and kidney function, and normal fibrinogen concentration. After discharge, dexamethasone and etoposide were used for continuous treatment. Follow-up within 2 years showed that the patient had a good status with negative plasma EBV DNA.

### Plasma Exosome Isolation and Characterization

**FIGURE 1:**
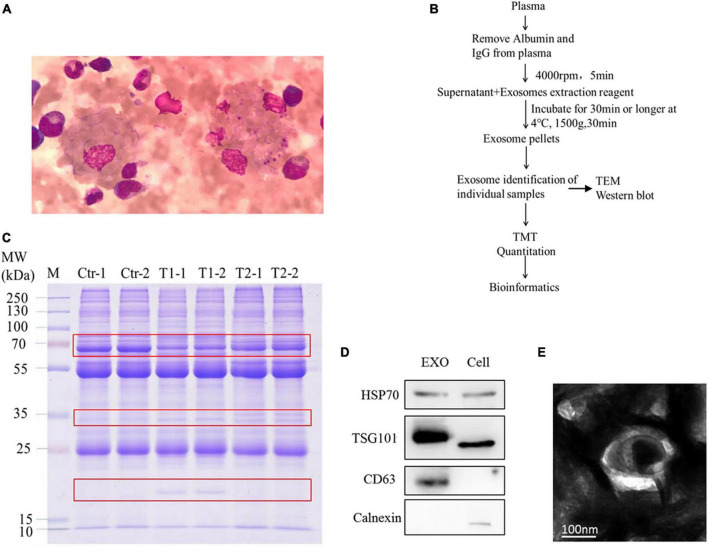
Bone marrow samples and plasma exosomal samples obtained from patients. **(A)** Wright–Giemsa staining of a smear of bone marrow aspirate shows hemophagocytic macrophages, including one with numerous platelets and red cells. **(B)** Overview of methodological approaches in high-throughput mass spectrometry-based proteomic analyses of extracellular vesicles. **(C)** Similar protein amounts (measured with the BCA assay) of six plasma exosome samples run on 4–20% gradient SDS-PAGE gels. The protein profile in the gel was visualized by Coomassie brilliant blue staining. **(D)** Western blot analysis of the positive exosome markers HSP70, TSG101, and CD63 and the negative exosome marker calnexin. **(E)** Transmission electron microscopy appearance of exosomes (bar, 100 nm).

In clinical detection, pleomorphic lymphocytes and hemophagocytosis were observed in the blood of the patient (Figure 1A). To ensure the quality of the TMT proteome, we used a series of methods to extract pure exosomes from plasma samples as shown in Figure 1B. The results of Coomassie brilliant blue staining showed that the removal of high-abundance proteins in plasma had a significant effect (Supplementary Figure 1), and a pattern of differentially expressed proteins was initially observed among the samples (Figure 1C). To verify the exosome extraction, we detected specific markers of exosomes using WB (Figure 1D). In addition, TEM analysis was applied to show the nanovesicle morphology (Figure 1E).

### Characteristics of the Plasma Exosomal Proteome and Protein–Protein Interaction Mapping

**FIGURE 2:**
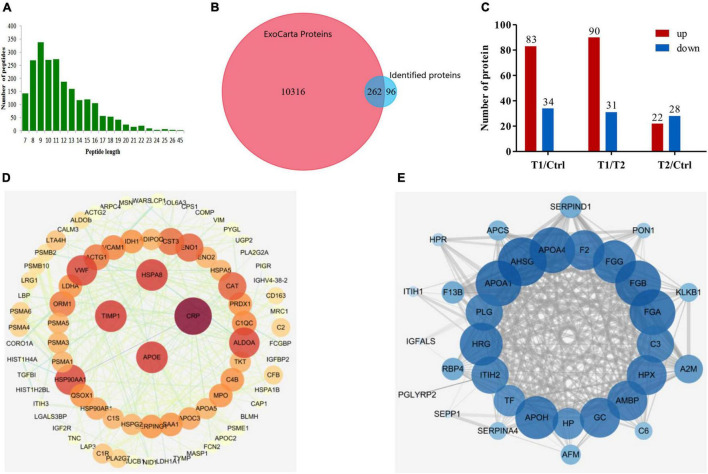
Characteristics of the plasma exosomal proteins and protein–protein interaction mapping. **(A)** Length distribution of peptides detected by LC-MS/MS after trypsin digestion. **(B)** Venn diagram presentation of overlaps and differences between identified exosomal proteins and the ExoCarta database. **(C)** Statistical information of differentially expressed proteins among the three groups of samples. **(D)** PPI network mapping of upregulated differentially expressed proteins in patients with EBV-HLH. **(E)** PPI network mapping of downregulated differentially expressed proteins in patients with EBV-HLH.

To determine the trypsin digestion efficiency of exosome proteins in this proteome, we statistically analyzed the average length and quantity distribution of the identified peptide sequences. As shown in Figure 2A, the peptides mainly had 8–13 amino acid residues, which indicates the high proteolytic efficiency of exosomes. TMT proteomics identified 358 proteins with genetic information. Among them, 262 proteins matched the exosome data previously published in the ExoCarta database, and the remaining 96 proteins might be newly identified exosomal proteins (Figure 2B). Thus, the identified proteins in this plasma exosome proteomic analysis had a 73% overlap with the exosome proteins published in the ExoCarta database, which indicates that the results of the exosome proteome are reliable. We used a difference of more than 1.5 times as the standard of significant upregulation and less than 1/1.5 as the standard of significant downregulation for intergroup comparison. Statistical analysis showed that 83 proteins had significantly upregulated expression and 34 proteins had significantly downregulated expression in the acute phase (T1 phase) when compared to those of the healthy control. However, after medical treatment (T2 phase), 90 proteins had significantly downregulated expression and 31 proteins had significantly upregulated expression when compared to those of the T1 phase. In addition, there are still some different proteins between the T2 phase and the healthy control (Figure 2C). Using the STRING database and Cytoscape tool, we presented the potential PPIs as shown in Figures 2D,E. We found that heat shock cognate 71 kDa protein (HSPA8), metalloproteinase inhibitor 1 (TIMP1), C-reactive protein (CRP), and apolipoprotein (APOE) proteins have the greatest interactions among those with upregulated expression in the T1 phase (Figure 2D). In addition, approximately 20 proteins including plasminogen (PLG) had the highest degree of interaction among the proteins with downregulated expression in the T1 phase (Figure 2E). These results indicated that all the above-mentioned proteins were among the hub molecules in the whole PPI network, which suggests their important role in the progress of the disease.

### Bioinformatic Analysis of Involved Functions and Pathways of the Differentially Expressed Proteins

**FIGURE 3:**
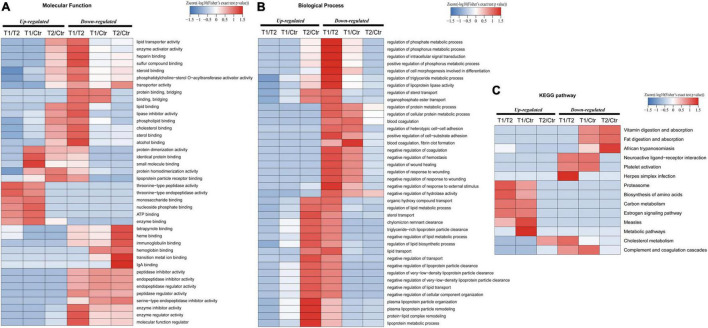
Gene ontology (GO) and kyoto encyclopedia of genes and genomes (KEGG) pathway enrichment analysis of differentially expressed proteins. **(A)** Molecular function enrichment analysis of different proteins among the T1, T2, and control groups. **(B)** Biological process enrichment analysis of the detected differentially expressed proteins among the T1, T2, and control groups. **(C)** Top 14 enriched KEGG pathways are referenced among the T1, T2, and control groups.

To further explore whether the exosome proteins were differentially expressed compared to the controls, we performed GO and KEGG pathway analyses to determine the significant enrichment trend of some special functions and related pathways (Figure 3). MF ontology indicated that the binding ability, enzyme activity, and inhibitor activity of these different proteins in the T1 phase were mostly disordered (Figure 3A). BP ontology indicated that in the T1 phase, various metabolic processes were enhanced, but the BPs related to “wound healing,” “coagulation,” and “cell adhesion” were significantly decreased (Figure 3B). KEGG pathway enrichment analysis showed that the T1 phase exhibited an increasing trend in the following pathways: “proteasome,” “biosynthesis of amino acids,” “carbon metabolism,” “estrogen signaling pathway,” and “measles” (Figure 3C). However, the decreased pathways in the T1 phase were mainly closely related to “vitamin digestion and absorption,” “fat digestion and absorption,” “African trypanosomiasis,” “neuroactive ligand-receptor interaction,” “platelet activation,” and “complement and coagulation cascades” (Figure 3C). Therefore, these main altered BPs and pathways detected in the plasma exosomal proteomes might play the important roles in the developmental process of EBV-HLH.

### Validation of Selected Proteins in Quantitative Proteomics

**FIGURE 4:**
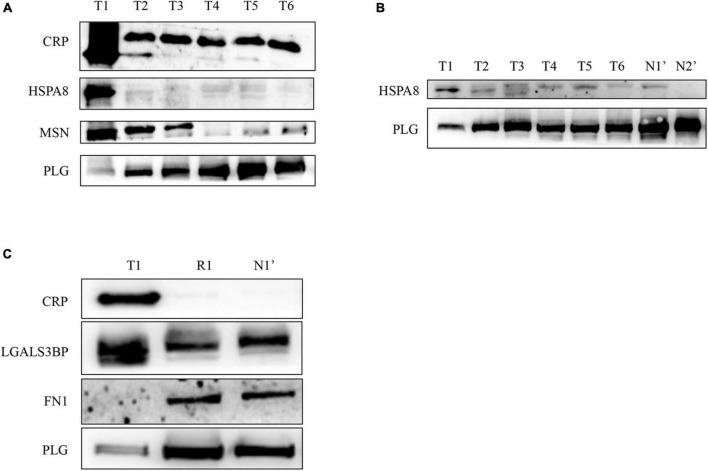
Verification of the differentially expressed proteins in plasma exosomes obtained from the patient with EBV-HLH by western blotting. **(A,B)** The expression levels of differentially expressed proteins during the treatment of the patient (T1–T6 represents the patient’s plasma exosome samples from the acute stage before treatment to 3 weeks after treatment and discharge, N1’ is the sample of his twin brother, N2’ is another healthy control sample). **(C)** The plasma exosomal protein expression level of the patient in the acute phase (T1) and 1 year after rehabilitation (R1) and that of the twin brother (N1’).

To validate the differentially expressed proteins identified by quantitative proteomics, we examined the expression of four proteins with upregulated expression [CRP, moesin (MSN), galectin 3-binding protein (LGALS3BP), and HSPA8] and two with downregulated expression [PLG and fibronectin 1 (FN1)] in different disease stages (T1–T6) of this patient by WB. As shown in Figure 4A, CRP and HSPA8 levels were significantly upregulated in the T1 phase and quickly decreased to a stable level after the treatment. In addition, the expression level of MSN gradually decreased with the progress of treatment. In addition, PLG expression was significantly downregulated in the T1 phase but gradually increased to a stable level as the treatment progressed (Figure 4A). Later, for the repeated verification of differentially expressed proteins, we extracted the plasma exosomes from the patient’s twin brother (N1’) and other healthy subjects (N2’) as controls. We found that the patient’s PLG and HSPA8 expression during T1–T6 stages gradually approached normal levels with the progress of treatment (Figure 4B). In addition, 1 year after discharge (R1), the patient’s plasma exosomal protein CRP, LGALS3BP, FN1, and PLG expression levels returned to normal level (N1’) (Figure 4C). All the results were consistent with those identified by mass spectrometry.

**FIGURE 5:**
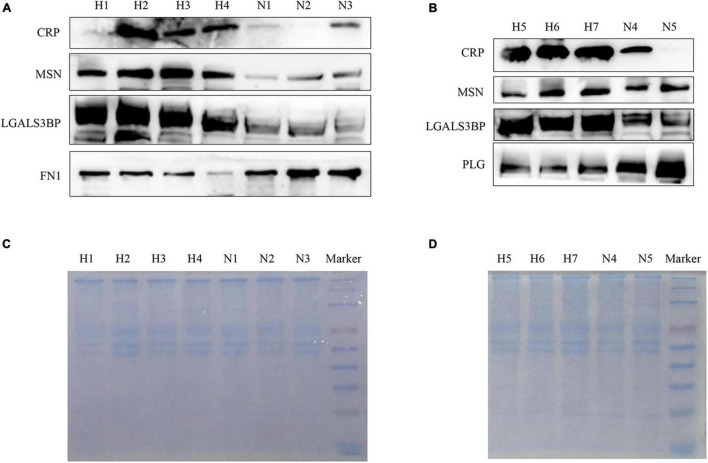
Verification of differentially expressed proteins in plasma exosomes obtained from other patients with EBV-HLH by western blotting. **(A,B)** The expression level of differentially expressed proteins in plasma exosomes between the patients with EBV-HLH and healthy controls (H represents patients with EBV-positive HLH, N represents healthy controls). **(C,D)** The total protein loading amount of each sample in **(A,B)** was visualized by Coomassie brilliant blue staining.

To further verify whether the mass spectrometry results have universal significance in EBV-positive HLH, we used plasma exosome samples from 14 other patients with EBV-positive HLH and 11 healthy controls for WB verification. The results showed that in the patients with EBV-positive HLH, the protein expression of CRP, MSN, LGALS3BP, and HSPA8 was upregulated, and FN1 and PLG levels were downregulated (Figures 5A,B and Supplementary Figure 2). The total protein loading control was showed by Coomassie brilliant blue staining as described in the previous literature (Kittivorapart et al., 2018; Liu Y. et al., 2020) (Figures 5C,D). These data indicated that the patient’s plasma exosomal proteomic analysis had general applicability for EBV-HLH.

**FIGURE 6:**
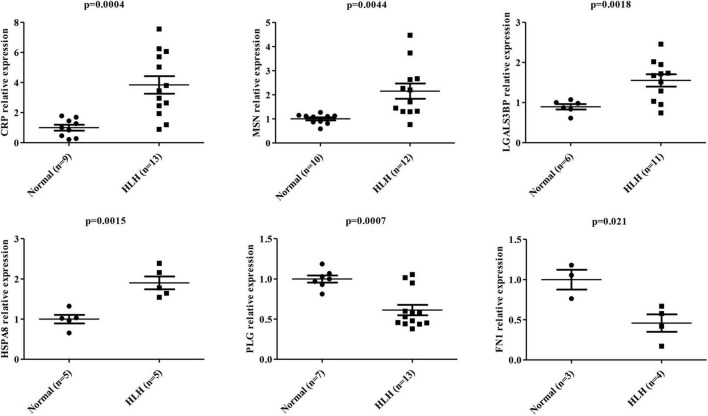
Statistical analysis of differentially expressed protein levels in plasma exosomes obtained from other patients with EBV-HLH. Gray value statistical analysis of differentially expressed protein levels of plasma exosomes between healthy controls (N) and other patients with EBV-positive HLH (H). A *t*-test was used to test the differences between groups.

According to the above WB results in Figure 5 and Supplementary Figure 2, a grayscale analysis for each identified molecule was performed. As shown in Figure 6, the patients with EBV-HLH had upregulated CRP levels (*p* = 0.0004), MSN levels (*p* = 0.0044), LGALS3BP levels (*p* = 0.0018), and HSPA8 levels (*p* = 0.0015) and downregulated PLG (*p* = 0.0007), and FN1 levels (*p* = 0.021) in the plasma exosomes.

## Discussion

Epstein-Barr virus-associated hemophagocytic lymphohistiocytosis occurs in children and adolescents, and occasionally in adults, which shows low remission rate, high recurrence rate, and mortality (Imashuku, 2002; Liu P. et al., 2020). Although spontaneous remission of EBV-HLH has been reported, it occurs in adults and is extremely rare (Chahine et al., 2020; Matsuo et al., 2021). The survival of patients with EBV-HLH is still worse (≥30% of patients die of the disease), and it is urgent to find the potential markers for early diagnosis and treatment (Meng et al., 2021). EBV-HLH could be classified as non-neoplastic and neoplastic, the former accompanies primary infection of EBV, and the latter accompanies EBV-positive lymphoid neoplasms, which includes EBV T/NK-cell lymphoma-associated HLH and CAEBV-associated HLH (El-Mallawany et al., 2022). These cases of EBV-HLH used in our study did not show obvious morphological atypia and lymphoid neoplasms, and these patients’ condition was improved significantly after HLH targeted treatment. Therefore, these cases used in this study belong to non-neoplastic EBV-HLH.

Exosome proteomics has been widely used to clarify the pathogenic mechanism of viral infection and explore the potential biomarkers of viral diseases, such as human immunodeficiency virus, SARS-CoV-2, and influenza A viruses (Cypryk et al., 2017; Kaddour et al., 2020; Barberis et al., 2021). Clinically, EBV-HLH cases are rare, so it is difficult to collect plasma samples. After a long-term storage, the early collected samples could lose some plasma-free protein. Exosomes could enrich intracellular substances and have membrane structure (Pegtel and Gould, 2019), which could avoid the degradation of their contents. In addition, our previous studies on nasopharyngeal carcinoma have shown that plasma exosomal proteins are more specific for disease diagnosis than plasma-free protein (Liu et al., 2019). Therefore, we choose exosomes for proteome research for EBV-HLH proteomics.

The EBV-HLH plasma exosomal proteome indicated that some interesting pathways were changed. EBV has been reported to manipulate the proteasome system to satisfy its own needs (Full et al., 2017), and also, the enhanced proteasome pathway in this patient with EBV-HLH may be associated with EBV infection. A previous article discussed whether it is measles or secondary HLH for a case diagnosis (Iaria et al., 2012; Lupo et al., 2012), which suggests some similar pathogenic mechanisms between EBV-HLH and measles. In addition, the measles signaling pathway is also significantly enriched in COVID-19 and systemic lupus erythematosus patients (Han et al., 2021; Gao et al., 2022), which indicates that there is a causal relationship between the enrichment of the measles signaling pathway and an overactive immune response. Therefore, the enhanced measles signaling pathway in patients with EBV-HLH may reflect a serious inflammatory state. The liver is a central organ of lipid metabolism (Nguyen et al., 2008), and its dysfunction is the typical clinical feature of EBV-HLH (Imashuku, 2002). Liang et al. (2017) revealed that a series of pathways, such as fat digestion and absorption, vitamin digestion and absorption, and African trypanosomiasis, could regulate lipid metabolism. In our study, these pathways were also downregulated in patients with EBV-HLH, which indicates defective lipid metabolism, thereby resulting in impaired liver function in patients with EBV-HLH. Coagulation disorder is also a common condition in EBV-HLH and is closely related to the high mortality rate of this disease (Valade et al., 2020, 2021). The occurrence of coagulation is mainly related to the function of platelets (Sang et al., 2021), so the decreased platelet activation pathway in this patient partially explains the mechanism of coagulopathy of EBV-HLH. Complement systems play a fundamental role in regulating immunity (Min et al., 2016), and the decreased complement and coagulation cascade pathways suggest an impaired immune response in patients with EBV-HLH. African trypanosomiasis, also known as sleeping sickness, is characterized by central nervous system (CNS) abnormalities in advanced patients (Kennedy, 2019). In addition, the estrogen signaling pathway and neuroactive ligand-receptor interaction pathway also regulate brain function and maintain the normal CNS (Crider and Pillai, 2017; Wei et al., 2020). Clinically, CNS diseases also occur in some patients with EBV-HLH (Imashuku, 2002). Therefore, the disorder in these three pathways could also lead to neurodevelopmental disorders, which may promote the progression of CNS diseases in EBV-HLH.

To confirm the validity of this proteomics, we verified the expression of six molecules (e.g., CRP, MSN, HSPA8, LGALS3BP, PLG, and FN1). These molecules have unique functions, and their abnormal expression may also explain the pathogenesis EBV-HLH and contribute to the early diagnosis of this disease. CRP, LGALS3BP, and HASPA8 have been shown to promote the innate immune response and inflammatory response and maintain cellular environmental homeostasis in viral infection (Xu et al., 2019; Herold et al., 2020; Wang et al., 2020; Luan et al., 2021). Therefore, the upregulated expression of these three molecules in EBV-HLH plasma exosomes may be an inflammatory indicator. MSN is involved in immune regulation (Serrador et al., 1997; Satooka et al., 2017), which especially enhances the phagocytic function of macrophages (Gomez and Descoteaux, 2018). However, an excessive expression of MSN may result in macrophage overactivation, which induces indiscriminate phagocytosis. In addition, PLG is a liver injury-specific protein (Raum et al., 1980) that plays an important role in maintaining normal liver function (Drixler et al., 2003; Wu et al., 2020). Therefore, downregulated PLG expression in plasma exosomes indicates impaired liver function in the patients with EBV-HLH. FN1 participates in blood coagulation and wound healing (Jaffe and Mosher, 1978; Mosher and Schad, 1979; Vaziri et al., 1993; Cai et al., 2018). Clinically, coagulation system disorders have been observed in most patients with EBV-HLH (Valade et al., 2020). Here, the downregulated FN1 in plasma exosomes of the patients with EBV-HLH could be the potential biomarker to indicate coagulation dysfunction.

In addition to the early diagnosis of EBV-HLH, the above potential biomarkers may provide some choices for clinical treatment. The four markers with upregulated expression (CRP, LGALS3BP, HSPA8, and MSN) are basically related to the immune dysregulation, and related inhibitors could be developed or used to inhibit the expression of these molecules, so as to promote the early anti-inflammatory and immune-modulating therapy for EBV-HLH. Moreover, engineered exosomes have been widely used to deliver certain drugs for disease treatment, such as the use of exosome-mediated TRIM3 delivery for gastric cancer (Fu et al., 2018) or miRNA-401 for anti-HSV-1 virus infection (Wang et al., 2018). The decreased expression of plasma exosomal PLG and FN1 in patients with EBV-HLH may result in an impairment of liver function and coagulation function. Therefore, exogenous supplementation of PLG and FN1 through engineered exosomes may also be a therapeutic approach to alleviate EBV-HLH.

It is difficult to collect EBV-HLH plasma samples in a short time clinically, but relatively fresh samples are required for proteomic analysis. Therefore, we only used this twin patient for mass spectrometry. Although the clinical sample size used for the validation was limited, the WB results were consistent with the proteomic analysis, and there were significant differences between the healthy controls and the patients with EBV-HLH, which may be because we selected the patient’s twin brother as a strict control for our proteomics. Therefore, our proteomic results could be universal in the clinic. In addition, the detection method of WB is troublesome for clinic. Enzyme-linked immunosorbent assay (ELISA) is convenient and fast, which has been used to detect those proteins in exosomes (Wang et al., 2021). Therefore, we think that ELISA could be a more appropriate method for the detection.

## Conclusion

In this study, we performed a high-throughput and in-depth analysis of the plasma exosomal proteomes of patients with EBV-HLH through quantitative proteomics. The twin samples showed high reliability for general application in clinical specimens. Several major altered pathways, which include proteasome, measles, platelet activation, complement and coagulation cascades, and some pathways related to lipid metabolism or nervous system regulation, were identified. Abnormalities in these pathways could be used to explain the clinical manifestations of EBV-HLH. In addition, our study revealed the overexpression of CRP, MSN, LGALS3BP, and HSPA8 and the downregulation of PLG and FN1 expression in the patients with EBV-HLH. These differentially expressed proteins could be the novel candidate biomarkers of EBV-HLH. However, to further determine the diagnostic and prognostic biomarkers, large-scale verification with clinical samples and follow-up assessment are required. Further study of the biological characteristics of these differentially expressed proteins will help clinical drug development.

## Data Availability Statement

All datasets presented in this study are included in the article/Supplementary Material. The mass spectrometry proteomics data have been deposited to the ProteomeXchange Consortium *via* the PRIDE (Perez-Riverol et al., 2019) partner repository with the dataset identifier PXD019088 and the project name: Plasma exosomal proteomic pattern in Hemophagocytic lymphohistiocytosis of a twin boy. The datasets used and/or analyzed during the current study are available from the corresponding author on request.

## Ethics Statement

The studies involving human participants were reviewed and approved by Medical Ethics Committee of Central South University. Written informed consent to participate in this study was provided by the participants’ legal guardian/next of kin. Written informed consent was obtained from the individual(s), and minor(s)’ legal guardian/next of kin, for the publication of any potentially identifiable images or data included in this article.

## Author Contributions

YX and LY contributed equally to this work, performed the experiments, and analyzed the data. YX, LY, and JHL designed and conceived the experiments. JHL supervised the research. YX, LY, and PC collected samples. PC, SL, WZ, WD, SX, MJ, and YJX collected samples and helped to interpret data. LY and JHL wrote and revised the manuscript. JL, SJL, YW, SZ, and YY assisted with the process of writing the manuscript. All authors read and approved the final manuscript.

## Conflict of Interest

The authors declare that the research was conducted in the absence of any commercial or financial relationships that could be construed as a potential conflict of interest.

## Publisher’s Note

All claims expressed in this article are solely those of the authors and do not necessarily represent those of their affiliated organizations, or those of the publisher, the editors and the reviewers. Any product that may be evaluated in this article, or claim that may be made by its manufacturer, is not guaranteed or endorsed by the publisher.

## Footnotes

1
www.http://www.ebi.ac.uk/GOA/

2
http://bioinformatics.psb.ugent.be/webtools/Venn/

## References

[B1] Al-Samkari H. Berliner N. (2018). Hemophagocytic lymphohistiocytosis. *Annu. Rev. Pathol.* 13 27–49. 10.1146/annurev-pathol-020117-043625 28934563

[B2] Barberis E. Vanella V. V. Falasca M. Caneapero V. Cappellano G. Raineri D. (2021). Circulating exosomes are strongly involved in SARS-CoV-2 infection. *Front. Mol. Biosci.* 8:632290. 10.3389/fmolb.2021.632290 33693030 PMC7937875

[B3] Berlot G. Tomasini A. Zandona L. Leonardo E. Bussani R. Zarrillo N. (2018). Fatal septic shock in a patient with hemophagocytic lymphohistiocytosis associated with an infectious mononucleosis. *Case Rep. Crit. Care* 2018:9756050. 10.1155/2018/9756050 30356381 PMC6176343

[B4] Cai X. Liu C. Zhang T. N. Zhu Y. W. Dong X. Xue P. (2018). Down-regulation of FN1 inhibits colorectal carcinogenesis by suppressing proliferation, migration, and invasion. *J. Cell. Biochem.* 119 4717–4728. 10.1002/jcb.26651 29274284

[B5] Chahine Z. Jayakrishnan T. Samhouri Y. Fazal S. (2020). Haemophagocytic lymphohistiocytosis that spontaneously resolved: a case of EBV. *BMJ Case Rep.* 13:e235544. 10.1136/bcr-2020-235544 33184051 PMC7662526

[B6] Crider A. Pillai A. (2017). Estrogen signaling as a therapeutic target in neurodevelopmental disorders. *J. Pharmacol. Exp. Ther.* 360 48–58. 10.1124/jpet.116.237412 27789681 PMC5193073

[B7] Cypryk W. Lorey M. Puustinen A. Nyman T. A. Matikainen S. (2017). Proteomic and bioinformatic characterization of extracellular vesicles released from human macrophages upon influenza a virus infection. *J. Proteome Res.* 16 217–227. 10.1021/acs.jproteome.6b00596 27723984

[B8] Drixler T. A. Vogten J. M. Gebbink M. F. Carmeliet P. Voest E. E. Borel Rinkes I. H. (2003). Plasminogen mediates liver regeneration and angiogenesis after experimental partial hepatectomy. *Br. J. Surg.* 90 1384–1390. 10.1002/bjs.4275 14598419

[B9] El-Mallawany N. K. Curry C. V. Allen C. E. (2022). Haemophagocytic lymphohistiocytosis and Epstein-Barr virus: a complex relationship with diverse origins, expression and outcomes. *Br. J. Haematol.* 196 31–44. 10.1111/bjh.17638 34169507

[B10] Filipovich A. H. Chandrakasan S. (2015). Pathogenesis of hemophagocytic lymphohistiocytosis. *Hematol. Oncol. Clin. North Am.* 29 895–902. 10.1016/j.hoc.2015.06.007 26461149

[B11] Fu H. Yang H. Zhang X. Wang B. Mao J. Li X. (2018). Exosomal TRIM3 is a novel marker and therapy target for gastric cancer. *J. Exp. Clin. Cancer Res.* 37:162. 10.1186/s13046-018-0825-0 30031392 PMC6054744

[B12] Full F. Hahn A. S. Großkopf A. K. Ensser A. (2017). Gammaherpesviral tegument proteins, PML-nuclear bodies and the ubiquitin-proteasome system. *Viruses* 9:308. 10.3390/v9100308 29065450 PMC5691659

[B13] Gao Z. Y. Su L. C. Wu Q. C. Sheng J. E. Wang Y. L. Dai Y. F. (2022). Bioinformatics analyses of gene expression profile identify key genes and functional pathways involved in cutaneous lupus Erythematosus. *Clin. Rheumatol.* 41 437–452. 10.1007/s10067-021-05913-2 34553293

[B14] Gomez C. P. Descoteaux A. (2018). Moesin and myosin IIA modulate phagolysosomal biogenesis in macrophages. *Biochem. Biophys. Res. Commun.* 495 1964–1971. 10.1016/j.bbrc.2017.12.061 29247647

[B15] Han K. Blair R. V. Iwanaga N. Liu F. Russell-Lodrigue K. E. Qin Z. (2021). Lung expression of human angiotensin-converting enzyme 2 sensitizes the mouse to SARS-CoV-2 infection. *Am. J. Respir. Cell Mol. Biol.* 64 79–88. 10.1165/rcmb.2020-0354OC 32991819 PMC7781002

[B16] Henter J.-I. Horne A. Aricó M. Egeler R. M. Filipovich A. H. Imashuku S. (2007). HLH-2004: diagnostic and therapeutic guidelines for hemophagocytic lymphohistiocytosis. *Pediatr. Blood Cancer* 48 124–131. 10.1002/pbc.21039 16937360

[B17] Herold T. Jurinovic V. Arnreich C. Lipworth B. J. Hellmuth J. C. Von Bergwelt-Baildon M. (2020). Elevated levels of IL-6 and CRP predict the need for mechanical ventilation in COVID-19. *J. Allergy Clin. Immunol.* 146 128–136.e124. 10.1016/j.jaci.2020.05.008 32425269 PMC7233239

[B18] Iaria C. Leonardi M. S. Buda A. Toro M. L. Cascio A. (2012). Measles and secondary hemophagocytic lymphohistiocytosis. *Emerg. Infect. Dis.* 18:1529; author reply 1529–1530. 10.3201/eid1809.120235 22931858 PMC3437716

[B19] Imashuku S. (2002). Clinical features and treatment strategies of Epstein-Barr virus-associated hemophagocytic lymphohistiocytosis. *Crit. Rev. Oncol. Hematol.* 44 259–272. 10.1016/s1040-8428(02)00117-8 12467966

[B20] Jaffe E. A. Mosher D. F. (1978). Synthesis of fibronectin by cultured human endothelial cells. *J. Exp. Med.* 147 1779–1791. 10.1084/jem.147.6.1779 355597 PMC2184307

[B21] Jordan M. B. Allen C. E. Weitzman S. Filipovich A. H. Mcclain K. L. (2011). How I treat hemophagocytic lymphohistiocytosis. *Blood* 118 4041–4052. 10.1182/blood-2011-03-278127 21828139 PMC3204727

[B22] Kaddour H. Lyu Y. Welch J. L. Paromov V. Mandape S. N. Sakhare S. S. (2020). Proteomics profiling of autologous blood and semen exosomes from HIV-infected and uninfected individuals reveals compositional and functional variabilities. *Mol. Cell. Proteomics* 19 78–100. 10.1074/mcp.RA119.001594 31676584 PMC6944229

[B23] Kennedy P. G. E. (2019). Update on human African trypanosomiasis (sleeping sickness). *J. Neurol.* 266 2334–2337. 10.1007/s00415-019-09425-7 31209574

[B24] Kittivorapart J. Crew V. K. Wilson M. C. Heesom K. J. Siritanaratkul N. Toye A. M. (2018). Quantitative proteomics of plasma vesicles identify novel biomarkers for hemoglobin E/β-thalassemic patients. *Blood Adv.* 2 95–104. 10.1182/bloodadvances.2017011726 29365317 PMC5787864

[B25] Ko Y. H. (2015). EBV and human cancer. *Exp. Mol. Med.* 47:e130. 10.1038/emm.2014.109 25613727 PMC4314581

[B26] Lee J. Y. Park J. K. Lee E. Y. Lee E. B. Song Y. W. (2016). Circulating exosomes from patients with systemic lupus erythematosus induce an proinflammatory immune response. *Arthritis Res. Ther.* 18:264. 10.1186/s13075-016-1159-y 27852323 PMC5112700

[B27] Liang P. Zhang M. Cheng W. Lin W. Chen L. (2017). Proteomic analysis of the effect of DHA-phospholipids from large yellow croaker roe on hyperlipidemic mice. *J. Agric. Food Chem.* 65 5107–5113. 10.1021/acs.jafc.7b00478 28438023

[B28] Liu L. Zuo L. Yang J. Xin S. Zhang J. Zhou J. (2019). Exosomal cyclophilin A as a novel noninvasive biomarker for Epstein-Barr virus associated nasopharyngeal carcinoma. *Cancer Med.* 8 3142–3151. 10.1002/cam4.2185 31063269 PMC6558463

[B29] Liu P. Pan X. Chen C. Niu T. Shuai X. Wang J. (2020). Nivolumab treatment of relapsed/refractory Epstein-Barr virus-associated hemophagocytic lymphohistiocytosis in adults. *Blood* 135 826–833. 10.1182/blood.2019003886 31914172

[B30] Liu Y. Fan J. Xu T. Ahmadinejad N. Hess K. Lin S. H. (2020). Extracellular vesicle tetraspanin-8 level predicts distant metastasis in non-small cell lung cancer after concurrent chemoradiation. *Sci. Adv.* 6:eaaz6162. 10.1126/sciadv.aaz6162 32195353 PMC7065889

[B31] Luan Y. Y. Yin C. H. Yao Y. M. (2021). Update advances on C-reactive protein in COVID-19 and other viral infections. *Front. Immunol.* 12:720363. 10.3389/fimmu.2021.720363 34447386 PMC8382792

[B32] Lupo J. Bernard S. Wintenberger C. Baccard M. Vabret A. Antona D. (2012). Fatal measles without rash in immunocompetent adult, France. *Emerg. Infect. Dis.* 18 521–523. 10.3201/eid1803.111300 22377448 PMC3309583

[B33] Marsh R. A. (2017). Epstein-Barr virus and hemophagocytic lymphohistiocytosis. *Front. Immunol.* 8:1902. 10.3389/fimmu.2017.01902 29358936 PMC5766650

[B34] Matsuo Y. Iwanami K. Hiraoka E. Oda R. (2021). Spontaneous recovery of hemophagocytic lymphohistiocytosis due to primary epstein-barr virus infection in an adult patient. *Am. J. Case Rep.* 22:e933272. 10.12659/ajcr.933272 34657119 PMC8532072

[B35] Meng G. Q. Wang J. S. Wang Y. N. Wei N. Wang Z. (2021). Rituximab-containing immuno-chemotherapy regimens are effective for the elimination of EBV for EBV-HLH with only and mainly B lymphocytes of EBV infection. *Int. Immunopharmacol.* 96:107606. 10.1016/j.intimp.2021.107606 33826999

[B36] Min L. Cheng J. Zhao S. Tian H. Zhang Y. Li S. (2016). Plasma-based proteomics reveals immune response, complement and coagulation cascades pathway shifts in heat-stressed lactating dairy cows. *J. Proteomics* 146 99–108. 10.1016/j.jprot.2016.06.008 27321583

[B37] Mosher D. F. Schad P. E. (1979). Cross-linking of fibronectin to collagen by blood coagulation Factor XIIIa. *J. Clin. Invest.* 64 781–787. 10.1172/jci109524 38260 PMC372182

[B38] Nguyen P. Leray V. Diez M. Serisier S. Le Bloc’h J. Siliart B. (2008). Liver lipid metabolism. *J. Anim. Physiol. Anim. Nutr. (Berl.)* 92 272–283. 10.1111/j.1439-0396.2007.00752.x 18477307

[B39] Pegtel D. M. Gould S. J. (2019). Exosomes. *Annu. Rev. Biochem.* 88 487–514. 31220978 10.1146/annurev-biochem-013118-111902

[B40] Perez-Riverol Y. Csordas A. Bai J. Bernal-Llinares M. Hewapathirana S. Kundu D. J. (2019). The PRIDE database and related tools and resources in 2019: improving support for quantification data. *Nucleic Acids Res.* 47 D442–D450. 10.1093/nar/gky1106 30395289 PMC6323896

[B41] Que Z. J. Luo B. Wang C. T. Qian F. F. Jiang Y. Li Y. (2020). Proteomics analysis of tumor exosomes reveals vital pathways of Jinfukang inhibiting circulating tumor cells metastasis in lung cancer. *J. Ethnopharmacol.* 256:112802. 10.1016/j.jep.2020.112802 32240782

[B42] Raum D. Marcus D. Alper C. A. Levey R. Taylor P. D. Starzl T. E. (1980). Synthesis of human plasminogen by the liver. *Science* 208 1036–1037. 10.1126/science.6990488 6990488 PMC2981173

[B43] Sang Y. Roest M. De Laat B. De Groot P. G. Huskens D. (2021). Interplay between platelets and coagulation. *Blood Rev.* 46:100733. 10.1016/j.blre.2020.100733 32682574 PMC7354275

[B44] Satooka H. Nagakubo D. Sato T. Hirata T. (2017). The ERM protein moesin regulates CD8(+) regulatory T cell homeostasis and self-tolerance. *J. Immunol.* 199 3418–3426. 10.4049/jimmunol.1700074 28978692

[B45] Serrador J. M. Alonso-Lebrero J. L. Del Pozo M. A. Furthmayr H. Schwartz-Albiez R. Calvo J. (1997). Moesin interacts with the cytoplasmic region of intercellular adhesion molecule-3 and is redistributed to the uropod of T lymphocytes during cell polarization. *J. Cell Biol.* 138 1409–1423. 10.1083/jcb.138.6.1409 9298994 PMC2132557

[B46] Song Y. Wang Y. Wang Z. (2019). Requirement for etoposide in the initial treatment of Epstein-Barr virus-associated haemophagocytic lymphohistiocytosis. *Br. J. Haematol.* 186 717–723. 10.1111/bjh.15988 31115044

[B47] Tabata YHibi S. Teramura T. Kuriyama K. Yagi T. Todo S. Sawada T. (2000). Molecular analysis of latent membrane protein 1 in patients with Epstein-Barr virus-associated hemophagocytic lymphohistiocytosis in Japan. *Leukemia Lymphoma* 38 373–380. 10.3109/10428190009087028 10830744

[B48] Valade S. Joly B. S. Veyradier A. Fadlallah J. Zafrani L. Lemiale V. (2021). Coagulation disorders in patients with severe hemophagocytic lymphohistiocytosis. *PLoS One* 16:e0251216. 10.1371/journal.pone.0251216 34343182 PMC8330932

[B49] Valade S. Mariotte E. Azoulay E. (2020). Coagulation disorders in hemophagocytic lymphohistiocytosis/macrophage activation syndrome. *Crit. Care Clin.* 36 415–426. 10.1016/j.ccc.2019.12.004 32172822

[B50] Vaziri N. D. Smith D. H. Winer R. L. Weber M. A. Gonzales E. C. Neutel J. M. (1993). Coagulation and inhibitory and fibrinolytic proteins in essential hypertension. *J. Am. Soc. Nephrol.* 4 222–228. 8400086 10.1681/ASN.V42222

[B51] Wang L. Chen X. Zhou X. Roizman B. Zhou G. G. (2018). miRNAs targeting ICP4 and delivered to susceptible cells in exosomes block HSV-1 replication in a dose-dependent manner. *Mol. Ther.* 26 1032–1039. 10.1016/j.ymthe.2018.02.016 29526650 PMC6080130

[B52] Wang L. Wu J. Song S. Chen H. Hu Y. Xu B. (2021). Plasma exosome-derived sentrin SUMO-specific protease 1: a prognostic biomarker in patients with osteosarcoma. *Front. Oncol.* 11:625109. 10.3389/fonc.2021.625109 33791211 PMC8006461

[B53] Wang Z. Li Y. Yang X. Zhao J. Cheng Y. Wang J. (2020). Mechanism and complex roles of HSC70 in viral infections. *Front. Microbiol.* 11:1577. 10.3389/fmicb.2020.01577 32849328 PMC7396710

[B54] Wei J. Liu J. Liang S. Sun M. Duan J. (2020). Low-dose exposure of silica nanoparticles induces neurotoxicity via neuroactive ligand-receptor interaction signaling pathway in zebrafish embryos. *Int. J. Nanomed.* 15 4407–4415. 10.2147/ijn.S254480 32606685 PMC7310985

[B55] Wu D. Zhang S. Xie Z. Chen E. Rao Q. Liu X. (2020). Plasminogen as a prognostic biomarker for HBV-related acute-on-chronic liver failure. *J. Clin. Invest.* 130 2069–2080. 10.1172/jci130197 32175919 PMC7108894

[B56] Xu G. Xia Z. Deng F. Liu L. Wang Q. Yu Y. (2019). Inducible LGALS3BP/90K activates antiviral innate immune responses by targeting TRAF6 and TRAF3 complex. *PLoS Pathog.* 15:e1008002. 10.1371/journal.ppat.1008002 31404116 PMC6705879

[B57] Zhang Q. Wei A. Ma H. H. Zhang L. Lian H. Y. Wang D. (2021). A pilot study of ruxolitinib as a front-line therapy for 12 children with secondary hemophagocytic lymphohistiocytosis. *Haematologica* 106 1892–1901. 10.3324/haematol.2020.253781 32732367 PMC8252948

