# Corrigendum: Plasma Exosomal Proteomic Pattern of Epstein-Barr Virus-Associated Hemophagocytic Lymphohistiocytosis

**DOI:** 10.3389/fmicb.2022.919026

**Published:** 2022-05-03

**Authors:** Yan Xie, Li Yang, Pengfei Cao, Shen Li, Wentao Zhang, Wei Dang, Shuyu Xin, Mingjuan Jiang, Yujie Xin, Jing Li, Sijing Long, Yiwei Wang, Senmiao Zhang, Yang Yang, Jianhong Lu

**Affiliations:** ^1^Department of Hematology, National Clinical Research Center for Geriatric Disorders, Xiangya Hospital, Central South University, Changsha, China; ^2^Department of Microbiology, School of Basic Medical Science, Central South University, Changsha, China; ^3^National Healthcare Commission (NHC) Key Laboratory of Carcinogenesis, The Key Laboratory of Carcinogenesis and Cancer Invasion of the Chinese Ministry of Education, Cancer Research Institute, Central South University, Changsha, China; ^4^China-Africa Research Center of Infectious Diseases, Central South University, Changsha, China

**Keywords:** hemophagocytic lymphohistiocytosis, Epstein-Barr virus, exosomes, quantitative proteomics, biomarker

In the published article, there was an error in affiliation ^**^1^**^. Instead of “^**^Department of Hematology, Xiangya Hospital, Central South University, Changsha, China^**^”, it should be “^**^Department of Hematology, National Clinical Research Center for Geriatric Disorders, Xiangya Hospital, Central South University, Changsha, China^**^”.

The authors apologize for this error and state that this does not change the scientific conclusions of the article in any way. The original article has been updated.

## Publisher's Note

All claims expressed in this article are solely those of the authors and do not necessarily represent those of their affiliated organizations, or those of the publisher, the editors and the reviewers. Any product that may be evaluated in this article, or claim that may be made by its manufacturer, is not guaranteed or endorsed by the publisher.

