# Supplementary material for: Plasma Exosomal Proteomic Pattern of Epstein-Barr Virus-Associated Hemophagocytic Lymphohistiocytosis
Source: Front Microbiol. 2022 Apr 6;13:821311. doi: 10.3389/fmicb.2022.821311 (PMC9019563; doi:10.3389/fmicb.2022.821311)
Supplement: Supplementary file 1 [file Table_1.DOCX]

**Table S1:** Antibodies used in this study

| Antibodies | Source | Identifier |
| --- | --- | --- |
| mouse anti-HSP70 | Proteintech | 66183-1- Ig |
| rabbit anti-TSG101 | Proteintech | 28283-1-AP |
| rabbit anti-CD63 | OriGene Technologies | TA802751 |
| rabbit anti-Calnexin | Abclonal | A4846 |
| rabbit anti-CRP | CST | 14316S |
| mouse anti-MSN | Santa Cruz | sc-13122 |
| rabbit anti-LGALS3BP | Proteintech | 10281-1AP |
| rabbit anti-HSPA8 | Abclonal | A14001 |
| rabbit anti-PLG | CST | 12657S |
| mouse anti-FN1 | SAB | 35542 |
| HRP Goat Anti-Mouse IgG (H+L) | Proteintech | PR30012 |
| HRP Goat Anti-Rabbit IgG (H+L) | Proteintech | PR30011 |
