# Supplementary figures and images for: Plasma Exosomal Proteomic Pattern of Epstein-Barr Virus-Associated Hemophagocytic Lymphohistiocytosis
Source: Front Microbiol. 2022 Apr 6;13:821311. doi: 10.3389/fmicb.2022.821311 (PMC9019563; doi:10.3389/fmicb.2022.821311)

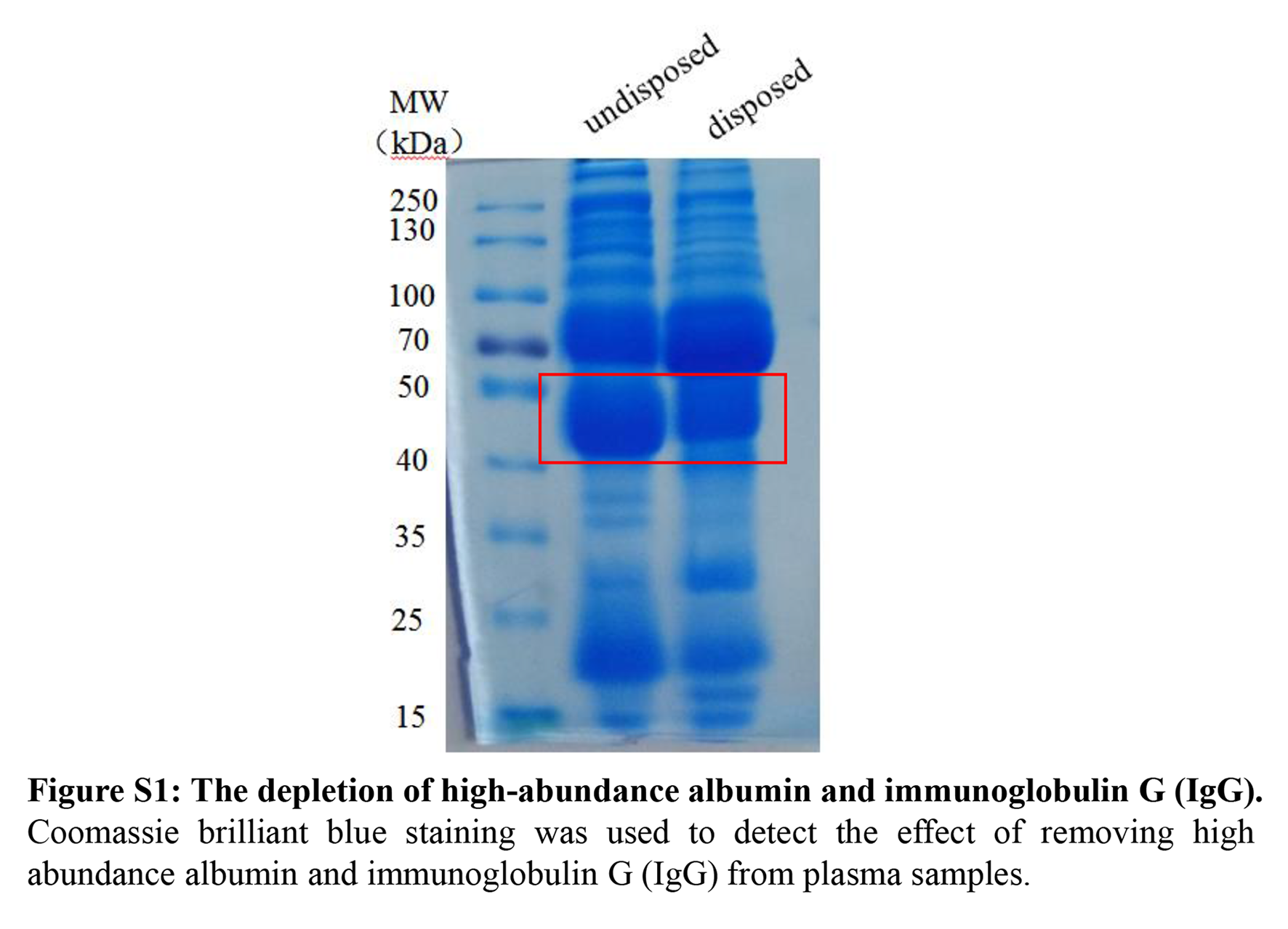

Supplement: Supplementary file 2 [file Image_1.TIF]

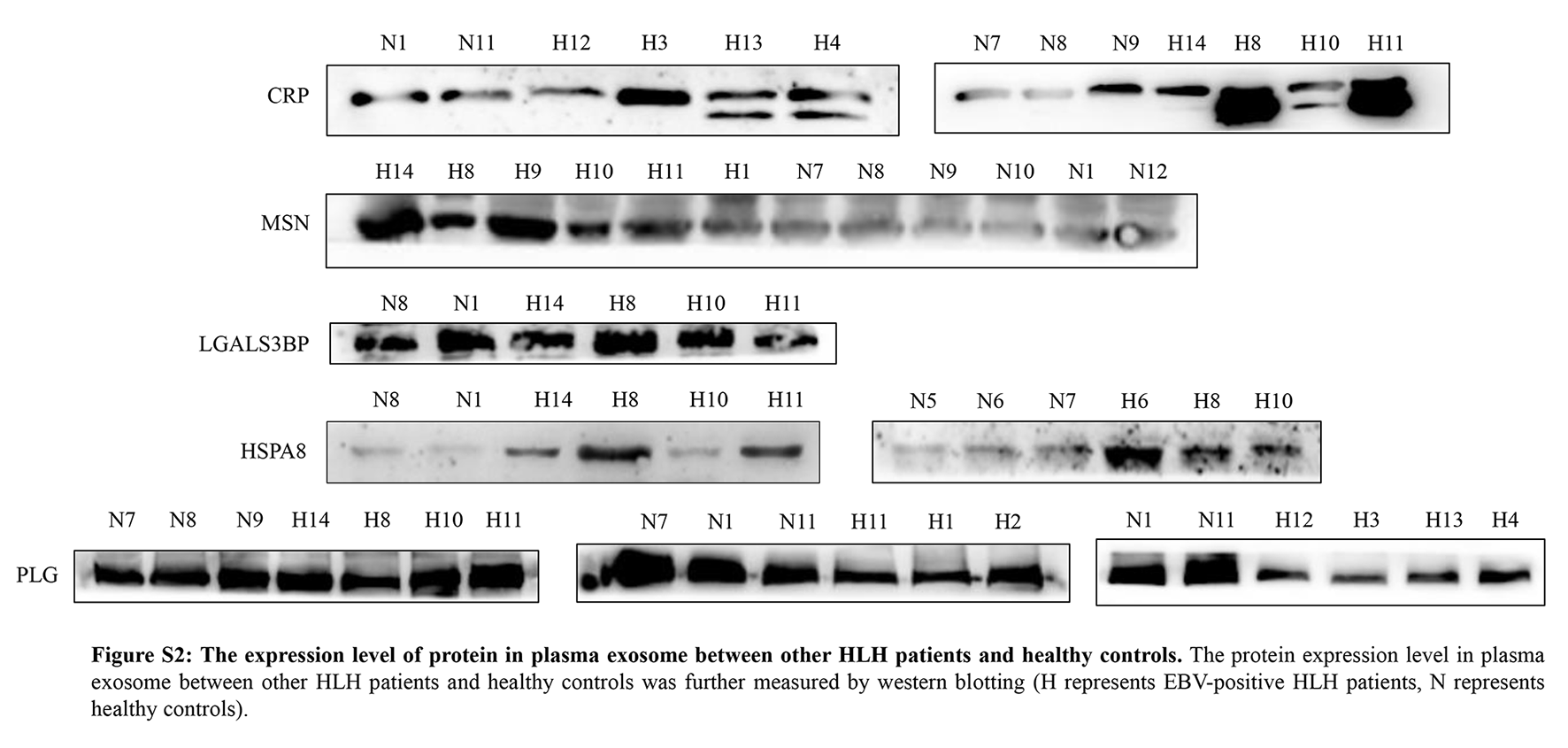

Supplement: Supplementary file 3 [file Image_2.TIF]
